# Intraoperative EMG recovery patterns and outcomes after RLN traction-related amplitude decrease during monitored thyroidectomy

**DOI:** 10.3389/fendo.2022.888381

**Published:** 2022-08-11

**Authors:** Kuan-Lin Chiu, Ching-Feng Lien, Chih-Chun Wang, Chien-Chung Wang, Tzer-Zen Hwang, Yu-Chen Shih, Wing-Hei Viola Yu, Che-Wei Wu, Gianlorenzo Dionigi, Tzu-Yen Huang, Feng-Yu Chiang

**Affiliations:** ^1^ Department of Otolaryngology-Head and Neck Surgery, E-Da Hospital, Kaohsiung, Taiwan; ^2^ School of Medicine, College of Medicine, I-Shou University, Kaohsiung, Taiwan; ^3^ Department of Otolaryngology, E-Da Cancer Hospital, Kaohsiung, Taiwan; ^4^ Department of Otorhinolaryngology-Head and Neck Surgery, International Thyroid Surgery Center, Kaohsiung Medical University Hospital, Faculty of Medicine, College of Medicine, Kaohsiung Medical University, Kaohsiung, Taiwan; ^5^ Division of General Surgery, Endocrine Surgery Section, Istituto Auxologico Italiano IRCCS, Milan, Italy; ^6^ Department of Pathophysiology and Transplantation, University of Milan, Milan, Italy

**Keywords:** recurrent laryngeal nerve (RLN), intraoperative neuromonitoring (IONM), thyroid surgery, electromyography (EMG), traction injury

## Abstract

**Objectives:**

Traction injury is the most common type of recurrent laryngeal nerve (RLN) injury in thyroid surgery. Intraoperative neuromonitoring (IONM) facilitates early detection of adverse electromyography (EMG) effect, and this corrective maneuver can reduce severe and repeated nerve injury. This study aimed to evaluate intraoperative patterns and outcomes of EMG decrease and recovery by traction injury.

**Methods:**

644 patients received nerve monitored thyroidectomy with 1142 RLNs at risk were enrolled. Intermittent IONM with stimulating dissecting instrument (real-time during surgical procedure) and trans-thyroid cartilage EMG recording method (without electrode malpositioning issue) were used for nerve stimulation and signal recording. When an EMG amplitude showed a decrease of >50% during RLN dissection, the surgical maneuver was paused immediately. Nerve dissection was restarted when the EMG amplitude was stable.

**Results:**

44/1142 (3.9%) RLNs exhibited a >50% EMG amplitude decrease during RLN dissection and all (100%) showed gradual progressive amplitude recovery within a few minutes after releasing thyroid traction (10 recovered from LOS; 34 recovered from a 51-90% amplitude decrease). Three EMG recovery patterns were noted, A-complete EMG recovery (n=14, 32%); B-incomplete EMG recovery with an injury point (n=16, 36%); C-incomplete EMG recovery without an injury point (n=14, 32%). Patients with postoperative weak or fixed vocal cord mobility in A, B, and C were 0(0%), 7(44%), and 2(14%), respectively. Complete EMG recovery was found in 14 nerves, and incomplete recovery was found in another 30 nerves. Temporary vocal cord palsy was found in 6 nerves due to unavoidable repeated traction.

**Conclusion:**

Early detection of traction-related RLN amplitude decrease allows monitoring of intraoperative EMG signal recovery during thyroid surgery. Different recovery patterns show different vocal cord function outcomes. To elucidate the recovery patterns can assist surgeons in the intraoperative decision making and postoperative management.

## Introduction

Routine identification of the recurrent laryngeal nerve (RLN) has been accepted as the gold standard of care during thyroid surgery to decrease RLN palsy rates (1–4). During the RLN identification and dissection phase, the RLN can be injured by the surgical maneuvers of traction, clamping, electrocauterization, mechanical trauma, and transection. However, traction injury is the most common cause of RLN injury, with a rate of up to 70-80% among all maneuvers (5–8). Medial traction of the thyroid lobe is a necessary step in the surgical procedure to identify RLN. The RLN can be overstretched and injured by a dense fibrous band or surrounding blood vessels at the region of Berry’s ligament during medial thyroid traction. Loss of signal (LOS) was found after nerve dissection with the application of intraoperative neuromonitoring (IONM) in thyroid surgery, even though visual anatomical integrity of the RLN was confirmed intraoperatively (8–10).

For stimulating methods, intermittent IONM (I-IONM) applied with a hand-held stimulating probe is still mainstream internationally due to its convenience and inexpensiveness. However, it is challenging to detect early between the intervals of nerve stimulation using the conventional I-IONM, the adverse electromyography (EMG) change, and LOS is consistently recognized after the occurrence of traction injury (5–8). Animal and clinical studies of RLN traction injury with the application of continuous IONM (C-IONM) (11–14) revealed an EMG amplitude decrease during RLN traction and progressive recovery after releasing traction stress. C-IONM is reported to be helpful for the early detection of EMG amplitude decreases caused by traction distress (15–17). Stimulating dissecting instrument (SDI) is a novel and real-time I-IONM stimulating method during surgical procedure. It can maximally reduce the intervals of the nerve stimulation while maintaining the advantages of I-IONM convenience, simplicity and affordability. For recording methods, unstable and variable EMG amplitudes during operation have been reported using traditional EMG tubes for EMG recoding (18, 19). It will be challenging to differentiate an actual decrease in EMG signal caused by traction stress on the RLN from a false decrease caused by EMG tube displacement. The trans-thyroid cartilage EMG recording method can avoid the electrode malpositioning issue and obtain high-quality EMG signals.

IONM facilitates early detection of adverse EMG effect, and the corrective maneuver can reduce severe and repeated nerve injury. However, intraoperative patterns and outcomes of EMG recovery are less studied in the literature. In this study, SDI and the trans-thyroid cartilage EMG recording method were used for nerve stimulation and signal recording. With these two new IONM techniques, we aimed to determine the feasibility of early detection of a significant EMG amplitude decrease during the phase of RLN dissection and the possibility of intraoperative EMG recovery after releasing thyroid traction, which might be helpful for avoiding signal deterioration to complete LOS and postoperative vocal cord (VC) palsy.

## Materials and methods

### Patients

**Figure 1:**
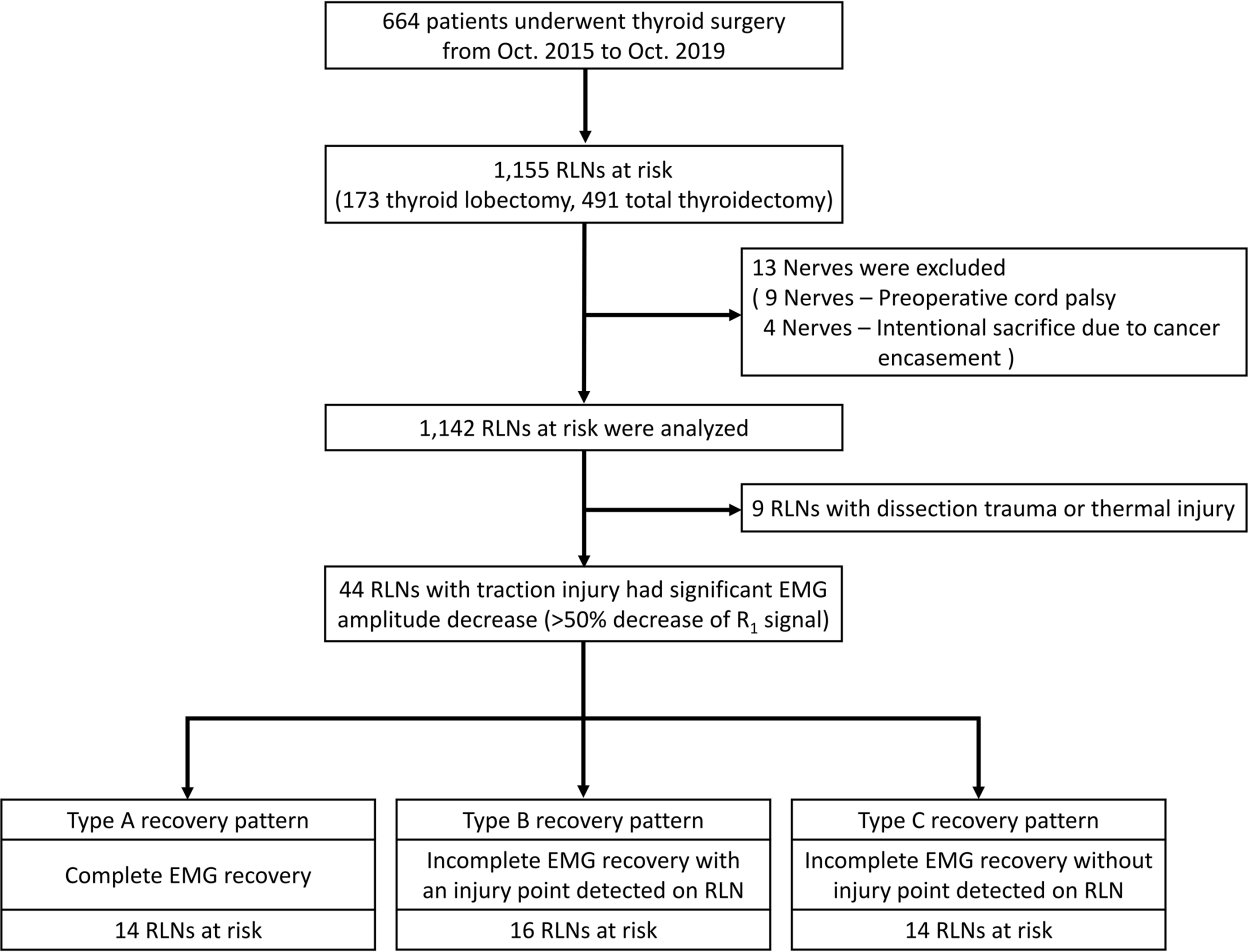
Flowchart of the procedure for the inclusion and exclusion of patients and the patterns, definitions, and patient distribution summarized from this study. RLN= Recurrent laryngeal nerve.

This retrospective study was conducted at a tertiary referral academic medical center, Kaohsiung Medical University Hospital, Taiwan. The data were collected from Oct. 2015 to Oct. 2019 regarding 664 patients (118 men and 546 women; ages ranging from 19 to 81 years; mean age, 50.9 years) who underwent operations for various thyroid diseases treated by the same surgeon (F.-Y. Chiang). There were 173 thyroid lobectomies and 491 total thyroidectomies (397 benign and 267 malignant thyroid diseases). Thirteen nerves were excluded from this study due to preoperative cord palsy (9 nerves) and intentional sacrifice due to cancer encasement (4 nerves). Thus, 1142 nerves at risk were enrolled in this study. (**Figure 1**)

### IONM setup and procedures

**Figure 2:**
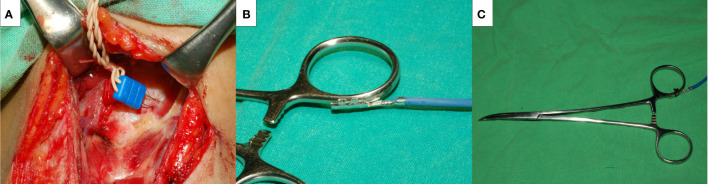
The novel intermittent intraoperative neuromonitoring method in this study. Medtronic two paired subdermal electrodes were inserted into the subperichondrium of the middle thyroid lamina for the EMG recording **(A)**. A Medtronic stimulation wire was connected to the conventional dissecting forceps **(B)**, and it became a stimulating dissecting instrument **(C)**.

General anesthesia was induced with lidocaine (1 mg/kg), propofol (2–3 mg/kg), a single dose of rocuronium (0.3 mg/kg), and a bolus of fentanyl (50 μg) as necessary. Regular oral endotracheal tubes were used for all patients. Anesthesia was maintained with sevoflurane and propofol target-controlled infusion. We routinely elevated the skin flap to the upper level of the thyroid cartilage and dissected the pyramidal lobe and prelaryngeal lymph nodes to place the electrodes correctly. Two paired subdermal electrodes (length, 12.0 mm; diameter, 0.4 mm; Medtronic, Jacksonville, FL) were inserted into the subperichondrium of the middle thyroid lamina for EMG recording (**Figure 2A**). The conventional dissecting forceps was connected by the stimulation wire to use it as SDI (**Figures 2B, C**), which was used for vagus nerve (VN) and RLN stimulation.

During the operation, standard IONM procedures (20) were strictly followed. The VN (without dissecting the carotid sheath to expose the VN) was stimulated with a 5-10 mA stimulus current before and after RLN dissection, and V_1_ and V_2_ signals were obtained. At the first identification, the RLN was stimulated with 3-5 mA, and the R1 signal was obtained. After complete RLN dissection, the exposed RLN was stimulated at the most proximal and distal ends near the laryngeal entry point, and R_2p_ and R_2d_ signals were obtained. The largest amplitudes of these five EMG signals (V_1_-R_1_-R_2p_-R_2d_-V_2_) were registered in all cases.

The EMG amplitude was monitored for changes during the phase of medial thyroid traction and RLN dissection. An EMG amplitude decrease >50% of the R_1_ signal was defined as a significant EMG amplitude decrease. When a significant EMG amplitude decrease was detected, the surgical maneuver was immediately stopped, and the mechanism (traction injury, thermal injury, and dissection trauma) of nerve injury was immediately determined and recovery of EMG amplitudes was closely monitored. Nerve dissection was restarted with gentle thyroid traction after the EMG amplitude recovered and reached a stable amplitude. A stable amplitude is defined as less than 10% amplitude change in one minute. Secondary significant EMG amplitude decreases can be observed and recorded in some nerves. After finishing RLN dissection, the amplitudes of R_2p_ and R_2d_ signals were compared. If the R_2p_/R_2d_ ratio reduction was over 10%, the whole exposed RLN was mapped to identify the injured point with 1 mA. The difference in the R_2p_ and R_2d_ signals within ±10% is regarded as the normal variation in the monitoring system. Before closing the wound, we repeated the same procedure to confirm and record the final data.

LOS was defined as the absence of EMG signals (amplitude less than 100 µV) after nerve stimulation. Type 1 LOS was defined as a localized injury point detected on the RLN, and Type 2 LOS was defined as no injury point on the whole exposed RLN. All patients received pre- and postoperative video recordings of VC function. Symmetric VC mobility was regarded as a normal VC function. Weak VC mobility was defined as asymmetric VC movement (VC still moved and was approximated well). VC palsy was defined as fixation of VC mobility. When VC palsy was found, VC function was examined every 2 weeks initially and every 4 weeks after that until complete recovery was achieved. VC palsy was considered permanent if it persisted for more than 6 months postoperatively.

The study was approved by the Institutional Review Board (IRB) of Kaohsiung Medical University Hospital (KMUHIRB-E(I)-20210358).

## Results

**Figure 3:**
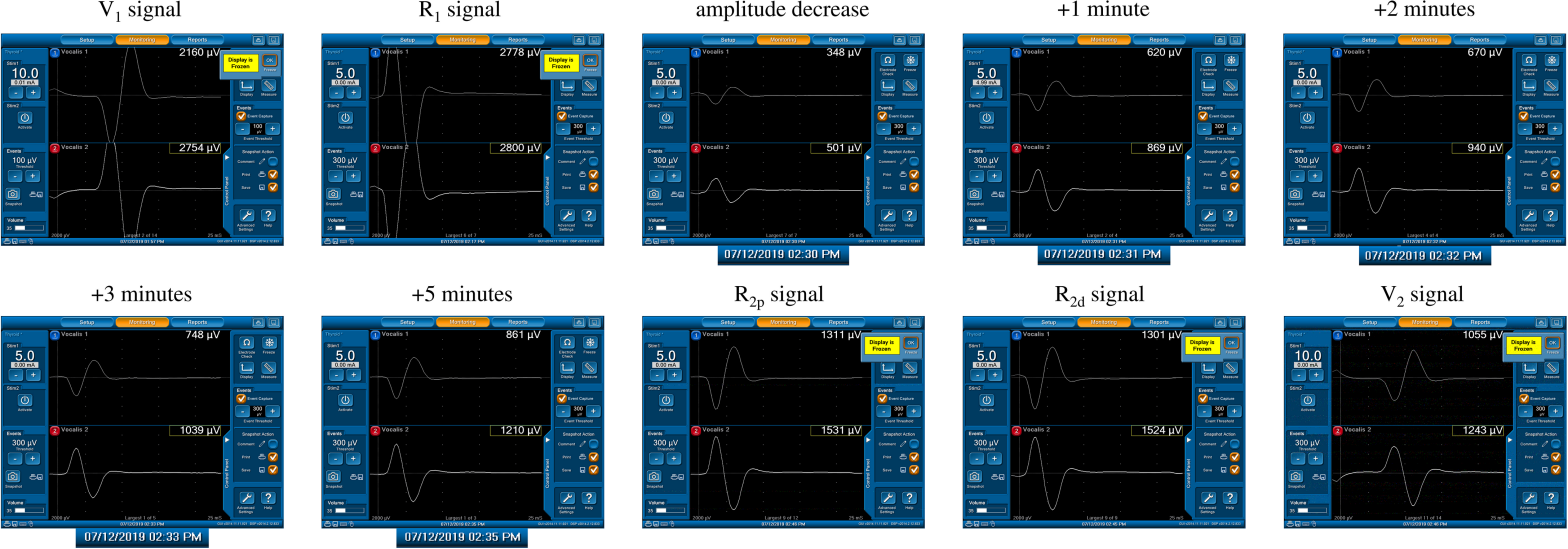
A significant amplitude decrease (channel 2) from 2800 µV (R1 signal) to 501 µV was detected. The actual operation time of the neuromonitoring screenshots is magnified and displayed below, according to amplitude decrease, +1, +2, +3, +5 minutes after amplitude decrease, respectively. Gradual and progressive amplitude recovery was found within a few minutes after releasing thyroid traction. The amplitudes between the R_2p_ and R_2d_ signals showed no difference, but there was a 66% amplitude decrease between the R_2p_ and R_1_ signals. This is a case of a Type C recovery pattern after traction injury.

Forty-four of 1142 nerves (3.9%) exhibited a significant EMG amplitude decrease (>50% decrease in R_1_ signal) during RLN dissection, and all (100%) showed gradual progressive amplitude recovery within a few minutes after releasing thyroid traction (**Figure 3**). Ten nerves recovered from LOS, and another 34 nerves recovered from a 51-90% amplitude decrease.

After restarting the surgical maneuver, a secondary significant EMG amplitude decrease occurred in 6 (13.6%) of 44 nerves. A substantial R_2p_/R_2d_ ratio reduction (81% and 92%) in 2 nerves and unrecovered LOS in 4 nerves occurred due to inevitable repeated traction, and all 6 nerves developed temporary VC palsy.

Three types of EMG recovery patterns were summarized. Type A recovery pattern showed complete EMG amplitude recovery in 14 (32%) nerves; Type B showed incomplete recovery with an injury point detected on the RLN in 16 (36%) nerves. Type C showed incomplete recovery without an injury point detected in 14 (32%) nerves (**Figure 1**). Regarding Type A recovery pattern, 2 of 14 nerves had LOS during the surgical maneuver. No (0%) patient had postoperative weak or fixed VC mobility (**Table 1**). Of the nerves with the Type B recovery pattern, 6 of 16 nerves had LOS during the surgical maneuver. Seven (44%) patients had abnormal postoperative VC mobility, including 2 patients had weak VC mobility and 5 patients had fixed VC mobility. All 16 nerve injury points were located in the upper portion of exposed RLN (at the region of Berry’s ligament or near the laryngeal entry point) (**Table 2**). Of the nerves with Type C recovery pattern, 2 of 14 nerves had LOS during the surgical maneuver. Two (14%) patients had abnormal postoperative VC mobility, including one patient had weak VC mobility, and one patient had fixed VC mobility (**Table 3**).

**Table 1:** Type A EMG recovery pattern (complete EMG recovery).

No.	V_1_(µV)	R_1_(µV)	R_2p_(µV)	R_2d_(µV)	V_2_(µV)	R_2p_/R_2d_ reduction^#^	Amplitude decrease (µV) ^+^	Amplitude decrease ratio (%)*	VC mobility
1	2532	3415	3276	3438	2322	nil	1565	54%	sym
2	1485	1944	1899	1944	1480	nil	535	72%	sym
3	1495	1661	1671	1681	1533	nil	276	83%	sym
4	1731	2159	2135	2131	1618	nil	559	74%	sym
5	853	1295	1177	1277	879	nil	353	73%	sym
6	1825	2490	2508	2525	1983	nil	452	82%	sym
7	785	772	833	881	767	nil	306	60%	sym
8	1397	1709	1652	1632	1240	nil	565	67%	sym
9	1875	1904	1988	2072	1711	nil	940	51%	sym
10	1223	1948	1859	1925	1334	nil	650	67%	sym
11	2624	3684	4229	4168	3024	nil	1565	58%	sym
12	2203	3350	3239	3345	2533	nil	819	63%	sym
13	1472	1805	1734	1751	1395	nil	LOS	≒100%	sym
14	1508	1880	1819	1801	1400	nil	LOS	≒100%	sym

**#** the difference of EMG signal between R_2p_ and R_2d_ within ±10% is regarded as a normal variation of the monitoring system.

**+** the decreased EMG signal detected during the surgical maneuver.

* the decrease ratio between the decreased EMG amplitude and R_1_ signal.

EMG, electromyography; VC, vocal cord; sym, symmetric.

**Table 2:** Type B EMG recovery pattern (incomplete EMG recovery with an injury point detected on RLN).

No.	V_1_(µV)	R_1_(µV)	R_2p_(µV)	R_2d_(µV)	V_2_(µV)	R_2p_/R_2d_ reduction^#^	Amplitude decrease (µV) ^+^	Amplitude decrease ratio (%)*	VC mobility
1	2862	2993	LOS	3279	LOS	≒100%	LOS	≒100%	fixed
2	1391	1581	LOS	1242	LOS	≒100%	LOS	≒100%	fixed
3	2430	2882	LOS	2976	LOS	≒100%	825	71%	fixed
4	3598	3752	1337	2806	1068	52%	LOS	≒100%	sym
5	2235	3695	1480	3855	1072	62%	LOS	≒100%	weak
6	1261	2222	512	1214	358	58%	LOS	≒100%	weak
7	1152	1289	145	755	138	81%	LOS	≒100%	fixed
8	2896	3773	323	3822	290	92%	1350	64%	fixed
9	1153	1278	364	1480	244	66%	429	66%	sym
10	2131	3278	1839	3307	1597	44%	1317	60%	sym
11	1213	1591	999	1274	893	22%	153	90%	sym
12	2716	3217	3075	2259	2096	36%	1323	59%	sym
13	2183	1913	1435	2054	811	30%	482	75%	sym
14	1957	2354	954	2325	801	59%	440	81%	sym
15	1289	1389	1182	1619	1101	28%	453	67%	sym
16	949	1017	708	1431	655	51%	305	70%	sym

The descriptions of #, +, *, and abbreviations are the same as those in **Table 1.**

**Table 3:** Type C EMG recovery pattern (incomplete EMG recovery without injury point detected on RLN).

No.	V_1_(µV)	R_1_(µV)	R_2p_(µV)	R_2d_(µV)	V_2_(µV)	R_2p_/R_2d_ reduction^#^	Amplitude decrease (µV) ^+^	Amplitude decrease ratio (%)*	VC mobility
1	1716	2585	LOS	LOS	LOS	nil	702	(73%)	fixed
2	1251	1515	509	462	346	nil-	LOS	≒100%	weak
3	1493	2331	1535	1516	912	nil	LOS	≒100%	sym
4	2845	3020	1553	1622	1628	nil	532	82%	sym
5	814	1422	852	872	414	nil	350	75%	sym
6	1014	1141	816	826	816	nil	406	64%	sym
7	2754	2800	1531	1524	1243	nil	501	82%	sym
8	511	784	346	362	254	nil	152	81%	sym
9	2490	2606	2260	2326	2166	nil	1135	56%	sym
10	1758	3221	2834	2807	2060	nil	1434	55%	sym
11	1675	2711	1907	2000	1528	nil	612	77%	sym
12	1905	3005	2586	2491	1837	nil	1350	55%	sym
13	2931	3385	2720	2626	2071	nil	1125	67%	sym
14	1715	2686	2226	2233	1510	nil	1154	57%	sym

The descriptions of #, +, *, and abbreviations are the same as those in **Table 1**.

In this study, a substantial EMG amplitude decrease was also found in 9 nerves caused by dissection trauma or thermal injury but without showing the feature of progressive recovery. Complete LOS was found in 5 nerves, and incomplete LOS was found in 4 nerves. Temporary VC palsy occurred in 7 nerves, and permanent VC palsy occurred in 2 nerve with thermal injury.

## Discussion

**Figure 4:**
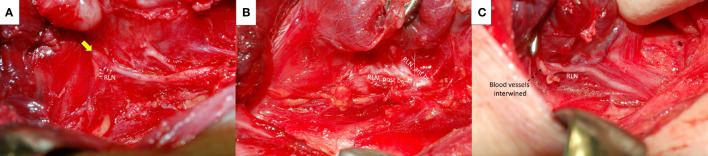
Recurrent laryngeal nerve (RLN) and Berry’s ligament. The RLN (white arrows) was severely stretched upward at the region of Berry’s ligament (yellow arrow) **(A)**. The anterior motor branch of the RLN penetrates Berry’s ligament **(B)**. A surrounding blood vessel intertwined (black dotted arrows) with the RLN at the region of Berry’s ligament **(C)**. ant br., anterior branch; post br., posterior branch.

RLN traction injury frequently occurs at the region of Berry’s ligament since the anatomical relationship between RLN, Berry’s ligament and surrounding vessels is highly variable (21–24) The RLN is located not only posterolateral to Berry’s ligament but also posteromedial to it, and the anterior motor branch of the RLN may penetrate the ligament. Additionally, blood vessels often intertwine with the RLN at the region of Berry’s ligament. The RLN can be overstretched during medial thyroid traction by Berry’s ligament or an intertwined vessel (**Figures 4A–C**).

When applying C-IONM in thyroid surgery, a progressive decrease in EMG amplitude is the characteristic feature of traction distress on the RLN (11–14). Prolonged traction may result in unrecovered LOS and postoperative VC palsy. Therefore, the weakness of conventional I-IONM compared to C-IONM is that early detection of imminent RLN injury and relief of traction distress are less effective to avoid signals deteriorating to an unrecoverable level (13). In this study, SDI was used for nerve stimulation and tissue dissection. It is a simple and effective way to real-time monitor nerve function during the highest risk phase for RLN injury (25, 26). In our experience, SDI is not inferior to C-IONM in early detection of nerve injury, furthermore, C-IONM cannot replace I-IONM in locating the nerve injury point. Additionally, we chose the trans-thyroid cartilage recording method for EMG signal recording. It provides high initial EMG amplitudes during VN and RLN stimulation, and the amplitudes remain stable during the entire course of the operation (27–29). This technique solves the major limitation of the EMG tube recording method. A significant EMG amplitude decrease can also occur due to EMG tube displacement during surgical manipulation of the thyroid lobe or trachea. Using an EMG tube for signal recording make it difficult to tell an actual EMG amplitude decrease from a false one. In this study, applying these two novel IONM techniques, we detected 44 nerves with a significant EMG amplitude decrease during RLN dissection and gradual progressive amplitude recovery after releasing thyroid traction. The EMG signal recovers from LOS in 10 nerves and a 51-90% amplitude decrease in 34 nerves. The results suggest a high chance of signal recovery after a substantial amplitude decrease, even after LOS, if traction strain on the RLN was relieved in time. In cases with large tumors or complicated anatomical relationships between the RLN and Berry’s ligament, a secondary EMG amplitude decrease still occurred after restarting the surgical maneuver with gentle thyroid traction and careful nerve dissection due to unavoidable repeated traction. A persistent substantial amplitude decrease was found in 6 nerves after finishing RLN dissection (2 nerves with 81% and 92% R_2p_/R_2d_ ratio reduction; 4 nerves with unrecovered LOS). All 6 nerves developed temporary VC palsy. The results indicate that the RLN is highly vulnerable to repetitive stretching.

From the results of the five EMG signals (V_1_-R_1_-R_2p_-R_2d_-V_2_) obtained from VN and RLN stimulation in this study, we found 3 types of EMG recovery patterns. Type A recovery showed complete EMG amplitude recovery, and the amplitudes of the R_2p_, R_2d_ and R_1_ signals were similar (**Table 1**). Type B recovery showed incomplete EMG recovery when comparing R_2p_ with R_1_ signals, and it was combined with an injury point on the RLN and a significant reduction in the R_2p_/R_2d_ ratio (**Table 2**). Type C recovery showed incomplete EMG recovery, but without an injury point detected on the RLN (**Table 3**). In Type C recovery pattern, we can see that the amplitudes of the R_2p_ and R_2d_ signals are similar, but the amplitudes show a significant decrease compared with the R_1_ signal (**Figure 3**). Case No. 2 in **Table 3** did not show a reduction of the R_2p_/R_2d_ ratio. Still, it had a 66% amplitude decrease compared to the R_2p_ and R_1_ signals (509 vs. 1515 µV), and this case developed weak VC mobility postoperatively. This kind of nerve injury was unrecognized when using the EMG tube recording method since the amplitude change would be mistaken for EMG tube displacement. Overall, different recovery patterns have different vocal cord function outcomes, to elucidate the recovery patterns can assist surgeons in the intraoperative decision making and postoperative management (i.e. speech therapy and injection laryngoplasty).

Regarding LOS after traction injury, Chiang (7) reported that nerve injuries caused by overstretching of Berry’s ligament could be divided into type 1 and type 2 stretch injuries. A type 1 stretch injury was caused by direct distress on the nerve and featured an injury point on the nerve. A type 2 stretch injury could be caused by pulling down the distal part of the RLN when the RLN was stretched excessively at the region of Berry’s ligament, and the nerve might be injured at a higher position above the laryngeal entry point. From the evidence of type B and type C EMG recovery patterns, we find that type 1 or type 2 nerve injury occurs not only in LOS (7, 9, 30) but also in incomplete LOS. In this study, there were 3 nerves with type 1 LOS and 1 nerve with type 2 LOS. In addition, there were 13 nerves with a type 1 incomplete LOS with a weak point of nerve conduction detected on the RLN (case No. 4-16 in **Table 2**) and 13 nerves with a type 2 incomplete LOS with no weak point detected (case No. 2-14 in **Table 3**).

In our clinical experience, some patients with very late recovery of muscle tone under neuromuscular blockade agent and the accuracy of the amplitudes of V1 and R1 may be questionable. In this study, the time from the administration of rocuronium (0.3 mg/kg), neck positioning, skin preparation to skin incision is around 20 minutes. And the time from skin incision, skin flap elevation, pyramidal dissection, superior thyroid pole dissection to V1 and R1 signals takes another 30 minutes, which is even longer. From the results of our patients without nerve injury, the data of V1-R1-R2p-R2d-V2 showed stable. It indicates that neuromuscular blockade has been almost completely recovered through 50 minutes. Therefore, the use of R1 signal as basic reference baseline should be reliable, although more studies are necessary to prove it.

Several limitations of this study were noted. First, this was an observational study and lacked a control group. Second, the comparisons between conventional I-IONM, SDI, and C-IONM were not studied. Furthermore, the combined SDI and C-IONM technique is an optimal procedure (least missed EMG changes) with distinct advantages that merit evaluation by a specific design study. Third, the recovery time might differ between type A, B, and C EMG recovery patterns in this study. When the nerve recovers quickly, the surgeon tends to wait and observe until the signal reaches a stable amplitude; when the nerve recovers slowly, the surgeon tends to perform surgical dissection of the contralateral or other regions first. Therefore, no exact recovery time can be provided in the current study, but a gentle thyroid traction and nerve dissection is strongly recommended after adverse EMG event. Fourth, the patient number of each recovery type is relatively small; however, the characteristics of each recovery type can be clearly shown in this study. Further large-scale studies in multiple centers are helpful for the analysis of the clinical presentation in the different recovery types.

## Conclusion

A significant EMG amplitude decrease and progressive recovery after releasing thyroid traction is the characteristic feature of traction strain on the RLN during thyroid surgery. In this study, early detection and release of thyroid traction results in intraoperative EMG recovery and helps to avoid a signal deterioration to an unrecoverable LOS. Gentle traction of the thyroid lobe is recommended during the entire course of the operation, and frequent stimulation of the RLN is necessary for the early detection of EMG amplitude changes, particularly when using a stimulating probe for nerve stimulation. Overall, different recovery patterns result in different vocal cord function outcomes which elucidate that the recovery patterns can assist surgeons in the intraoperative decision making and postoperative management.

## Data availability statement

The original contributions presented in the study are included in the article. Further inquiries can be directed to the corresponding authors.

## Ethics statement

Ethical approval of this study was obtained from the Kaohsiung Medical University Hospital Institutional Review Board (KMUHIRB-E(I)-20210358). Written informed consent for participation was not required for this study in accordance with the national legislation and the institutional requirements.

## Author contributions

Supervision – T-ZH, W-HY, C-WW, GD, and F-YC; Materials – C-FL, T-ZH, Y-CS, T-YH, and F-YC; Data Collection and Processing – K-LC, ChihCW, ChieCW , and Y-CS; Analysis and Interpretation – K-LC, C-FL, W-HVY, T-YH, and F-YC; Literature Search – K-LC, ChihCW, ChieCW , T-YH, and F-YC; Writing Manuscript – All authors. All authors have read and agreed to the published version of the manuscript.

## Funding

This study was supported by grants from Kaohsiung Medical University Hospital, Kaohsiung Medical University (KMUH110-0R51), and Ministry of Science and Technology (MOST 110-2314-B-037-104-MY2, MOST 110-2314-B-037-120, MOST 111-2628-B-037-007), Taiwan.

## Acknowledgments

The authors would like to thank all the patients included in this study.

## Conflict of interest

The authors declare that the research was conducted in the absence of any commercial or financial relationships that could be construed as a potential conflict of interest.

## Publisher’s note

All claims expressed in this article are solely those of the authors and do not necessarily represent those of their affiliated organizations, or those of the publisher, the editors and the reviewers. Any product that may be evaluated in this article, or claim that may be made by its manufacturer, is not guaranteed or endorsed by the publisher.
